# Desulfurization and Denitrification Performance of Modified Rice Husk Ash-Carbide Slag Absorbent

**DOI:** 10.3390/ma14010068

**Published:** 2020-12-25

**Authors:** Yali Wang, Xiaoning Han, Meina Chen, Suping Cui, Xiaoyu Ma, Liwei Hao

**Affiliations:** 1 Faculty of Materials and Manufacturing, Beijing University of Technology, Beijing 100124, China; hanxiaoning@emails.bjut.edu.cn (X.H.); chenmeina@emails.bjut.edu.cn (M.C.); cuisuping@bjut.edu.cn (S.C.); maxiaoyu@bjut.edu.cn (X.M.); 2 State Key Laboratory of Solid Waste Reuse for Building Materials, Beijing Building Materials Academy of Science Research, Beijing 100041, China; haoliwei@bbma.com.cn

**Keywords:** rice husk ash, carbide slag, hydration, modification, desulfurization and denitrification

## Abstract

In the cement industry, SO_2_ and NOx are generally removed separately. There are many problems, such as large area, high investment cost, secondary pollution and so on. Desulfurization and denitrification technology have become a frontier research direction in the field of air pollution control. In this paper, rice husk ash and carbide slag were compounded and modified to prepare modified rice husk ash-carbide slag composite absorbent, and its desulfurization and denitrification performance and mechanism were studied. The results showed that the NO conversion and SO_2_ conversion of the modified rice husk ash-carbide slag composite absorbent increased by 44% and 2%, respectively, at 700 °C. Fibrous calcium silicate and calcium silicoaluminate hydrates were formed during the hydration process, which made the specific surface area of the absorbent larger and provided more reactive sites. The hydration process increases the content of oxygen-containing functional groups, decreases the hydroxyl/ether C–O functional groups, and increases the content of carboxyl–COO functional groups are conducive to the denitrification reaction.

## 1. Introduction

SO_2_ and NOx are the main atmospheric pollutants in the flue gas of cement industry kilns, which can cause severe acid rain [1]. In the cement industry, it is common to remove SO_2_ and NOx separately. Integrated technology can be divided into combined and simultaneous desulfurization and denitrification technology [2]. A combination of wet flue gas desulfurization technology and selective catalytic reduction technology for NOx removal is generally adopted [3]. The integrated flue gas desulfurization and denitrification technology can achieve effective desulfurization and denitrification in the same system, streamlined equipment, convenient operation and management, and has become a frontier research direction in the field of air pollution control [4]. The dry reactant absorption method is currently the most concerned method, which can simultaneously treat a kind of waste gas containing SO_2_ and NO. Dry-process absorbents include calcium-based absorbents, calcium-based fly ash absorbents, natural manganese ore, activated carbon, activated carbon impregnated catalysts and metal catalysts. In order to synthesize an absorbent with high desulfurization and denitrification activity, silicon raw materials usually need to be activated by CaO or Ca(OH)_2_. Most of the siliceous materials mentioned in the paper are fly ash [5,6,7,8,9] and oil palm ash [10], diatomite and silica fume [11,12]. In recent years, rice husk ash has been found to have great potential as a silicon source material due to its large amount of amorphous SiO_2_ [13].

Dahlan et al. [3] used rice husk ash and CaO to synthesize absorbent by hydration method, which can completely remove SO_2_ at 100 °C. Lee [14] found that the absorbent of rice husk ash impregnated with copper oxide can effectively remove both SO_2_ and NOx in a flue gas environment at 150 °C. The denitrification mechanism of calcium-based absorbent is to use alkaline substances to convert NOx in flue gas into nitrate or nitrite. In this process, the actual denitrification object is NO_2_, but more than 90% of the nitrogen oxides in the flue gas are NO. Therefore, the prerequisite for achieving denitrification is to first oxidize the NO in the flue gas to NO_2_ before entering the reactor or during the reaction. The desulfurization and denitrification efficiency of calcium-based absorbent needs to be further improved, especially the low denitrification efficiency, which has become an important bottleneck restricting the popularization of this technology in the market [15]. The desulfurization and denitrification absorbent currently studied in the experiment is generally used at a lower temperature around 100 °C, and usually plays a role in humidified flue gas environments and is not suitable for cement processes. Therefore, there is no good integrated desulfurization and denitrification method that can be implemented in the cement industry, and the traditional separate removal technology is still used.

Studies have shown that CaO is a highly active substance catalyzing C–NO reaction. CaO and C reactions will generate CaC_2_, which can reduce the emission of NO in a reducing atmosphere [16]. Carbide slag is the waste residue produced during the preparation of acetylene. Its main components are Ca(OH)_2_ and CaCO_3_, as well as a small amount of MgO, SiO_2_, Al_2_O_3_ and Fe_2_O_3_ [17]. Carbide slag is a strong alkaline slurry with a fine particle size (particles of 10–50 µm account for about 80%), and its properties are similar to those of slaked lime milk, with a pH of over 12 [18]. As carbide slag is rich in CaO, the combination of carbide slag and rice husk ash may play a synergistic role in desulfurization and denitrification, thus achieving a better effect. In order to synthesize an absorbent with high desulfurization and denitrification activity, silicon raw materials usually need to be activated by CaO or Ca(OH)_2_. Rice husk ash has been found to have great potential as a silicon source material due to its large amount of amorphous SiO_2_.

In our previous experiments, we explored the properties of absorbents prepared from rice husk ash and carbide slag to simultaneously remove SO_2_ and NO. The combination of rice husk ash biomass charcoal reduction of nitrogen oxides and calcium hydroxide fixation of calcium hydroxide in carbide slag can effectively desulfurize and denitrify. However, the optimal sulfur fixation temperature is 500–600 °C in cement kilns, while the reaction temperature range of C and NOx is 750–900 °C. The SO_2_ and NOx emission reduction temperature ranges are not equal, and there is also a phenomenon of competitive adsorption. High desulfurization and denitrification effect can only be achieved under high-temperature conditions of 900 °C [19]. In order to broaden the reaction temperature window, we modified the rice husk ash-carbide slag absorbent by changing the hydration temperature, hydration time, rice husk ash/carbide slag mass ratio, and solid-liquid mass ratio. A kind of highly active absorbent was prepared to strengthen the coupling of C–CaO–SiO_2_ under the working condition of the cement kiln.

Explore the hydration mechanism of rice husk ash and carbide slag, establish the component control, particle surface modification and surface activation methods of rice husk ash- carbide slag desulfurization and denitrification absorbent, and propose a set of high-efficiency and energy-saving new solutions for desulfurization and denitrification in the cement industry. With the increasing pressure of environmental protection, the development of a highly active absorbent for dry desulfurization and denitrification without ammonia under the working conditions of cement kilns has a good application prospect.

## 2. Experiment and Measurement Methods

### 2.1. Characterization of Materials

The carbide slag used in this paper was from a chemical plant, and the rice husk ash was from a waste resource development and utilization Co., LTD. The ash content of rice husk ash and the chemical composition of carbide slag were analyzed by X-ray fluorescence spectrometer (XRF-1800, made by Shimadzu, Tokyo, Japan), and the results are shown in Table 1.

The measurement of their specific surface area, pore size distribution and pore volume using the BET method, using nitrogen gas adsorption, measured by high-speed automatic specific surface area and porosity analyzer (TriStar Ⅱ 3020, made by Micromeritics, Norcross, GA, USA). The sample phase analysis was performed using X-ray diffractometer (XRD-7000, made by Shimadzu, Tokyo, Japan). The surface microscopic morphology and structure were detected using SEM (SU8020, made by Hitachi High-tech, Tokyo, Japan). Analysis of surface element composition and valence of materials were detected by X-ray Photoelectron Spectroscopy (XPS, Escalab 250Xi, made by Thermo Fisher, Waltham, MA, USA).

### 2.2. Desulfurization and Denitrification Performance Test System

**Figure 1:**
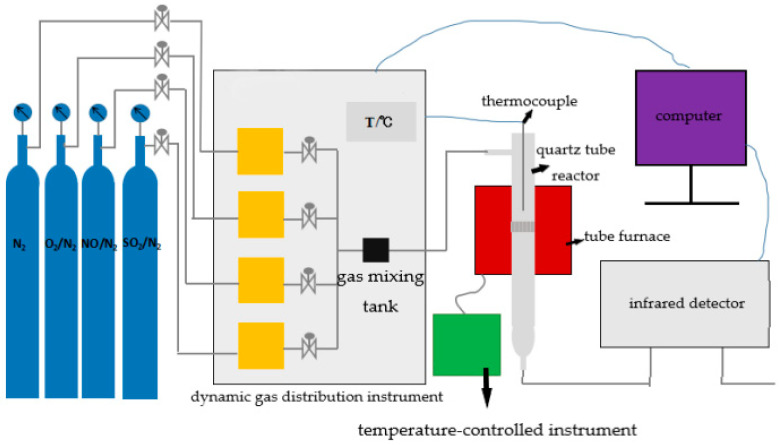
Simultaneous reaction and test device for desulfurization and denitrification.

The desulfurization and denitrification performance test system mainly consist of air source, dynamic gas distribution instrument, reactor and Fourier transform infrared spectrometer. The gas sources are NO/N_2_, SO_2_/N_2_, N_2_ and O_2_/N_2_, which are used to simulate the flue gas composition of the cement kiln. The dynamic gas distribution apparatus simulates the flue gas conditions of different concentrations by adjusting the flow of different gases. The reactor consists of a quartz tube and a tube furnace with controlled temperature. The test sample was placed on the grid in the middle of the quartz tube, carried by quartz cotton, and reacted with the gas at high temperature. The concentrations of NOx and SO_2_ in the atmosphere before and after the reaction were measured by the Fourier transform infrared spectrometer. The structure of the whole device is shown in Figure 1.

The absorbent was mixed into the cement raw meal at a mass ratio of 10%, and the simultaneous removal experiment of SO_2_ and NO was carried out under simulated flue gas conditions. The temperature of flue gas is in the range of 300–900 °C, O_2_ concentration is 1%, NO concentration is 1000 ppm, SO_2_ concentration is 2000 ppm, and the rest is N_2_ as the balance gas. The gas flow rate is 450 mL/min.

The denitrification and desulfurization performance is described by the NO conversion and the SO_2_ conversion.

The calculation formula of NO conversion is:

η_1_ = (1 − [NO_x_]_out_/[NO_x_]_in_) × 100%. (1)

The calculation formula of SO_2_ conversion is:

η_2_ = (1 − [SO_2_]_out_/[SO_2_]_in_) × 100%. (2)

In the equation: η_1_ represents the NO conversion, [NO_X_]_in_ and [NO_X_]_out_ represent the inlet and outlet concentration of NO. η_2_ represents the SO_2_ conversion, [SO_2_]_in_ and [SO_2_]_out_ represent the inlet and outlet concentration of SO_2_.

### 2.3. Experimental Method for Modification of Rice Husk Ash-Carbide Slag Sbsorbent

The rice husk ash-carbide slag absorbent was modified by the hydration method. By changing the hydration temperature, hydration time, rice husk ash/ carbide slag mass ratio and solid–liquid mass ratio, absorbents with different desulfurization and denitrification performance are obtained, nine groups of different modification conditions were designed to prepare modified absorbent, as shown in Table 2.

**Figure 2:**
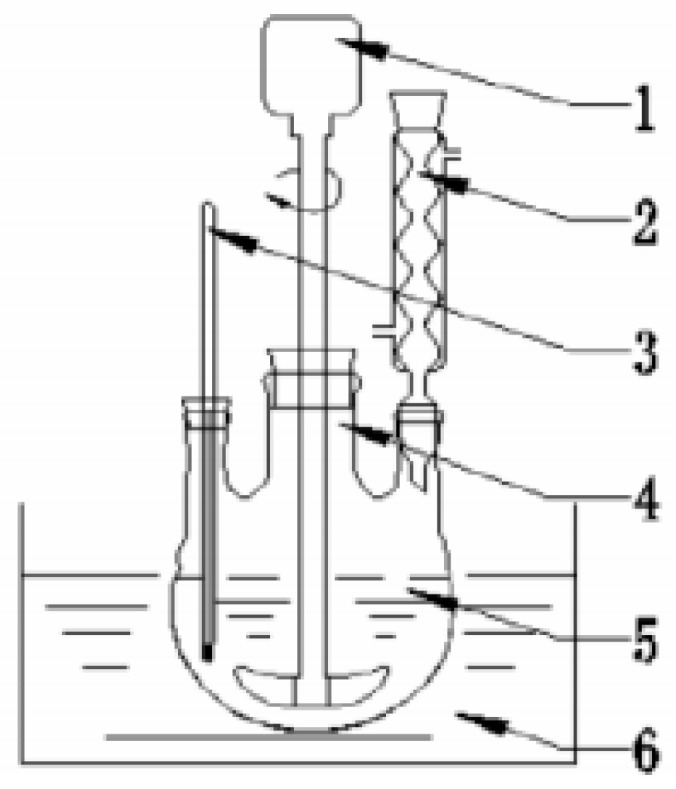
Experimental reaction device for absorbent modification: 1—stirring equipment; 2—condenser; 3—temperature; 4—blender; 5—three-necked flask; 6—oil bath.

The modification methods of rice husk ash-carbide slag absorbent are as follows:

1. Rice husk ash and carbide slag with a certain mass ratio were weighed and a certain amount of deionized water was measured.
2. After the deionized water is heated to 60–70 °C, put carbide slag into the water and continuously stirred. After the mixture is evenly mixed, the temperature is raised to 70–85 °C, and the rice husk ash is added into the slurry. After that, the temperature is raised to the required hydration temperature and the magnetic stirring is maintained at 300–500 r/min to promote the hydration reaction. The experimental device is shown in Figure 2.
3. When the predetermined hydration time is reached, the absorbent slurry is cooled and then pumped for dehydration. Dry the absorbent at 150 °C for 2 h. After drying, grind the fine powder into 100 mesh sieve, and put it into a wide-mouth bottle for airtight storage.

## 3. Results and Discussion

### 3.1. Desulfurization and Denitrification Performance of Modified Absorbent

#### 3.1.1. The Effect of Modification Conditions on the Performance of Absorbent

**Figure 3:**
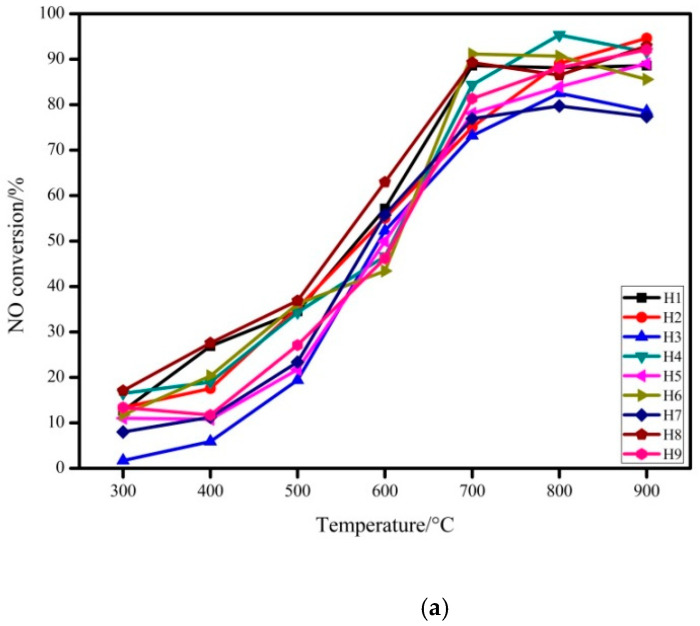

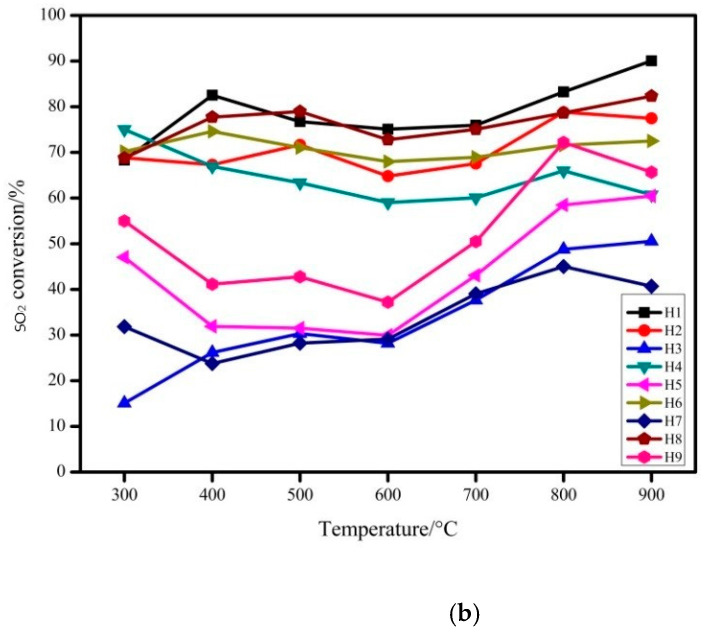
Desulfurization and denitrification performances of modified absorbents in the full temperature range (300–900 °C): (**a**) NO conversion as a function of the temperature (300–900 °C) of the modified absorbents and (**b**) SO_2_ conversion as a function of the temperature (300–900 °C) of the modified absorbents.

The denitrification and desulfurization performance of the absorbent modified by hydration treatment are tested in the full temperature range of 300–900 °C, such as in Figure 3.

From Figure 3, 9 groups of absorbents showed higher NO conversion at the beginning of 700 °C, and increases slowly after 700 °C. In the temperature range of 600–700 °C, the NO conversion increases the most. The SO_2_ conversion fluctuates slightly in the whole temperature range. The optimal condition is: the hydration temperature is 70 °C, the hydration time is 8 h, the mass ratio of rice husk ash/carbide slag is 1:1, the mass ratio of solid-liquid is 1:20. When the hydration time is 8 h, the mass ratio is 1:1, and the solid-liquid ratio is 1:20, the desulfurization performance and denitrification performance are the best. For the hydration temperature, 130 °C is the best under the comprehensive analysis of the two indicators. The hydration reaction of rice husk ash and carbide slag is a relatively slow process. The formation of hydration products requires the absorption of heat. Therefore, the higher temperature will promote the formation of products and increase the specific surface area. At the same time, sufficient hydration time can make the reaction more complete. However, too-long hydration time will cause the decomposition of calcium aluminosilicate product, and increase the cost from a practical industrial point of view. As the mass ratio of rice husk ash/carbide slag increases, the desulfurization and denitrification performance of the absorbent decreases. The higher the carbide slag content, the more Ca^2+^ in the slurry participates in the formation of hydrated aluminosilicate. The hydration reaction is a very complex physical and chemical process, which is affected by factors such as the pH value of the solution, the concentration of the solution, and the number of particles dissolved in rice husk ash and carbide slag. The pH of the slurry is increased when the solid–liquid ratio is too high, but the excessive solid content prevents the effective components in the rice husk ash and carbide slag from being fully dissolved, which is not conducive to the reaction; if the solid-liquid ratio is too small, excess water will dilute the mixed slurry. Lowering the pH of the solution is not conducive to the formation of reaction products [20]. Therefore, it is determined that the optimal preparation condition of the absorbent is: the hydration temperature is 130 °C, the hydration time is 8 h, the mass ratio of rice husk ash/ carbide slag is 1:1, and the solid-liquid ratio is 1:20, namely sample H8.

#### 3.1.2. Desulfurization and Denitrification Performance of Absorbent before and after Modification

**Figure 4:**
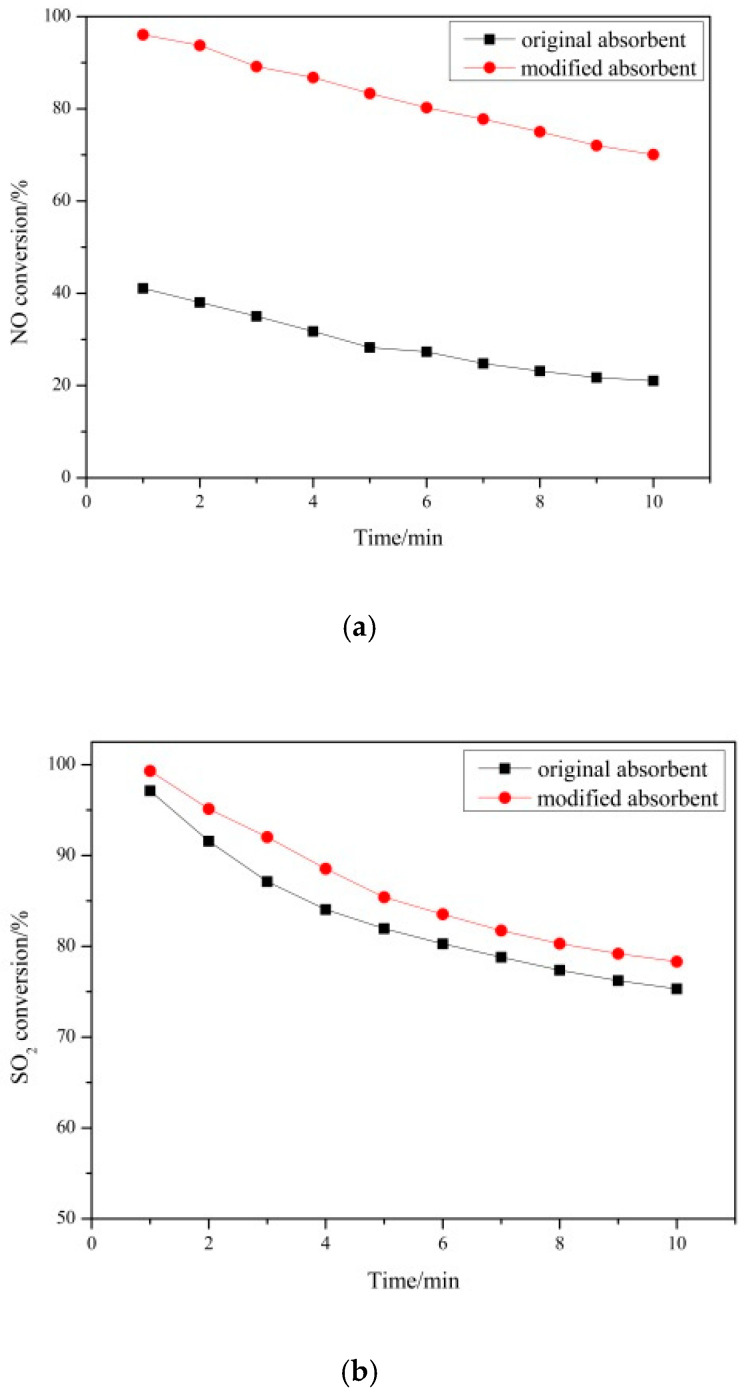
Desulfurization and denitrification performance before and after modification of absorbents: (**a**) NO conversion as a function of the time of the original absorbent and modified absorbent and (**b**) SO_2_ conversion as a function of the time of the original absorbent and modified absorbent.

The absorbent before and after the modification was added to the cement raw meal at a mass ratio of 10%. The comparison of the desulfurization and denitrification performance at 700°C is shown in Figure 4. The NO conversion of the absorbent before modification is 41%, and the SO_2_ conversion is 97%; the NO conversion of the modified absorbent reaches 96%, and the SO_2_ conversion reaches 99%. The NO conversion is greatly improved, which is an increase of 44%. The conversion of change is small, with an increase of about 2%. After modification, the desulfurization performance of the absorbent has little change. It can be seen that the abundant Ca(OH)_2_ in carbide slag is the main reason for the desulfurization effect, and the modification process increases the denitrification effect of the absorbent.

### 3.2. Composition and Structure of Modified Absorbent

#### 3.2.1. BET Results

The specific surface area analysis of rice husk ash, carbide slag and modified absorbent is shown in Table 3. It can be seen that rice husk ash has a larger specific surface area and a smaller pore size than carbide slag. The modified absorbent has a larger specific surface area and a large number of pore structures ranging from several nanometers to several tens of microns. This is because rice husk ash and carbide slag form the reactive substances during the hydration process, which provide more places for the denitrification reaction and expand the temperature window of the denitrification reaction.

#### 3.2.2. Phase Analysis

**Figure 5:**
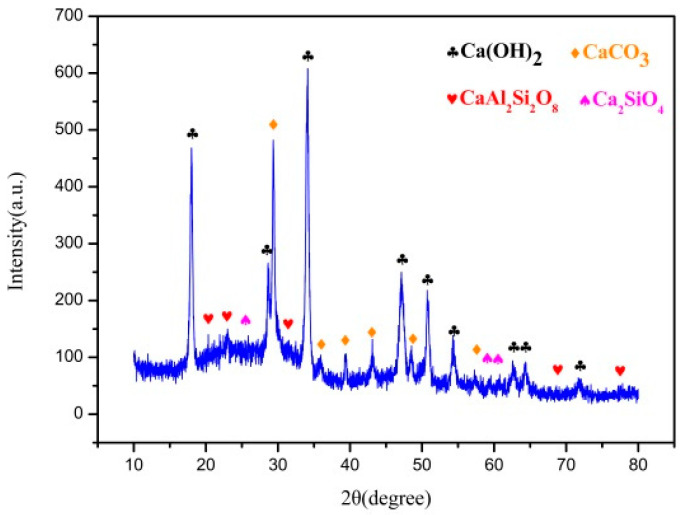
XRD pattern of modified absorbent.

Figure 5 is the XRD pattern of modified absorbent. The carbide slag is mainly Ca(OH)_2_ phase and a small amount of CaCO_3_ phase. In addition to CaCO_3_, Ca(OH)_2_ and amorphous SiO_2_ phases, CaAl_2_Si_2_O_8_ and Ca_2_SiO_4_ were also found in the modified absorbent. The hydration modification process causes the CaO and metal oxides in the carbide slag to react with rice husk ash to generate new active substances (hydrated calcium silicate and calcium aluminosilicate). At the same time, the absorbent modified by hydration has a larger specific surface area, provides more reaction surface for the reaction, and is more conducive to the C–NO reaction.

#### 3.2.3. Shape Analysis

**Figure 6:**
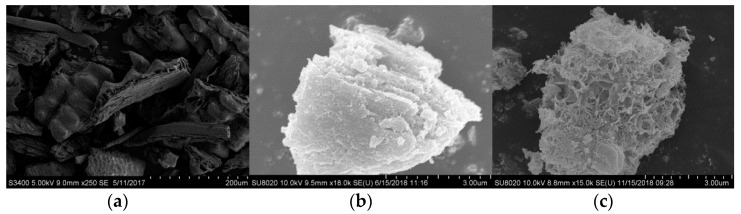
Scanning electron microscope of rice husk ash, carbide slag and modified absorbent: (**a**) rice hush ask, (**b**) carbide slag and (**c**) modified absorbent.

Figure 6a–c is scanning electron micrographs of rice husk ash, carbide slag and modified absorbent, respectively. It can be seen from the figure that the rice husk ash is divided into three layers of upper, middle and lower layers, and the interlayer is composed of crisscrossed plates, forming a loose honeycomb porous structure. There is a dense SiO_2_ protective film on the inner and outer surfaces. The surface of carbide slag is lamellar without an obvious pore structure. The modified absorbent not only has an obvious pore structure, but also has flocs on the surface. It is the active substance produced in the hydration process of rice husk ash and carbide slag–fibrous silicate hydrate. It has a specific surface area much larger than Ca(OH)_2_.

#### 3.2.4. XPS Analysis

**Figure 7:**
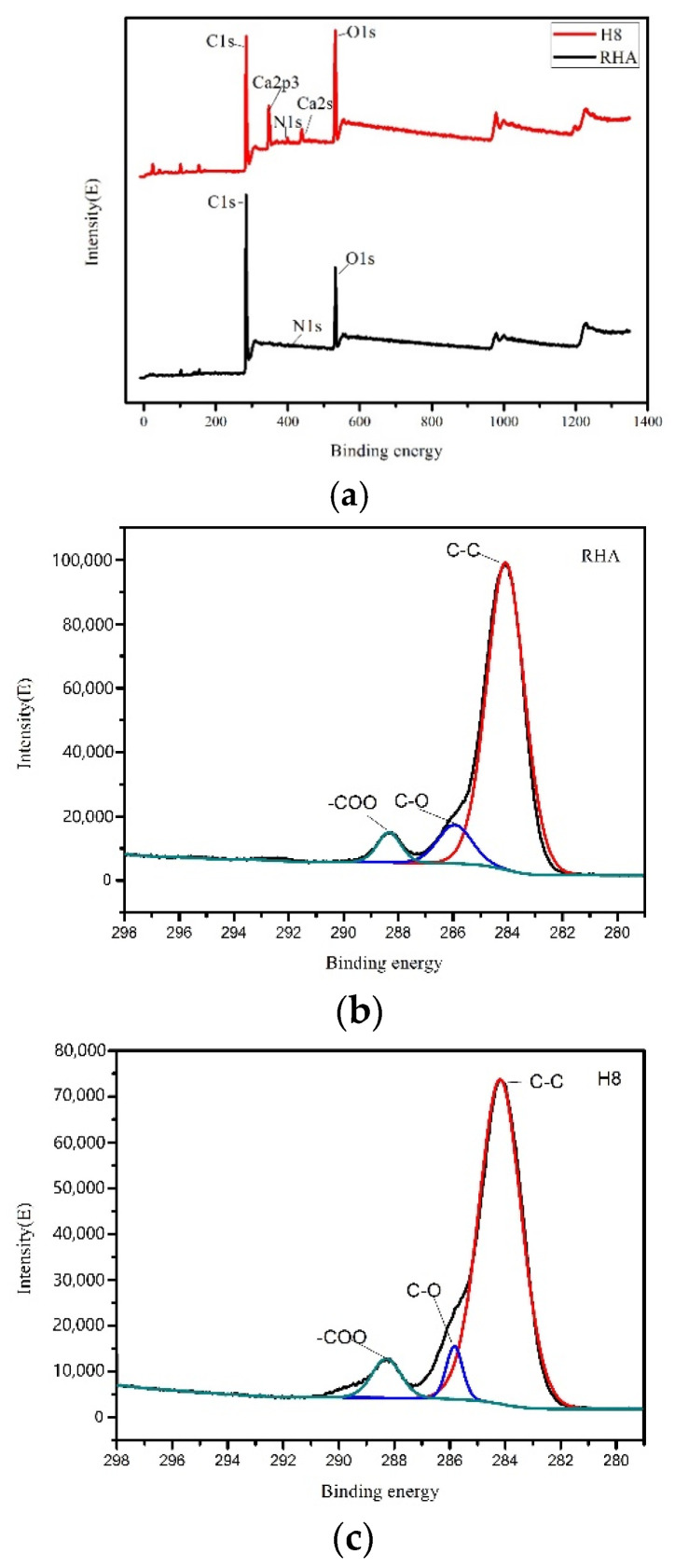
Photoelectron spectroscopy contrast diagram of RHA and H8: (**a**) Photoelectron spectroscopy of RHA and H8; (**b**) XPS fitting peaks of RHA and (**c**) XPS fitting peaks of H8.

Compared with rice husk ash, the denitrification effect of the modified absorbent has been greatly changed. Therefore, XPS was used to analyze the surface chemical properties of the modified absorbent and rice husk ash. The XPS measurement spectra of the modified absorbent and rice husk ash showed that C, N and O were mainly present on the surface. The relative contents of these elements are listed in Table 4. The N content of the modified absorbent has a slight change, with an increase from 0.58% to 2.05%; the C content has a greater change compared to rice husk ash, from 82.62% to 70.09%; the O content is significantly increased from 16.80% to 27.86%, the O/C element ratio increased from 0.2 (rice husk ash) to 0.4. This indicates that the hydration process increases the content of oxygen-containing functional groups. Figure 7 shows the photoelectron spectra of rice husk ash and modified absorbent H8. The C1s peak in the energy spectrum of rice husk ash was fitted to obtain three characteristic peaks, including C, N, and O; When fitting the C1s peak in the energy spectrum of the modified absorbent, there are a total of 4 characteristic peaks, in addition to C, N, O, it also includes Ca.

Table 5 lists the relative content of each functional group. XPS cannot distinguish between ether groups and hydroxyl groups, so C–O is taken as the sum of ether groups and hydroxyl groups during analysis. The results showed that the number of graphitized carbon C–C and hydroxyl/ether C–O decreased to 84.10% and 5.33%, respectively. The carboxy–COO content increased to 6.98%. The carboxy–COO is believed to facilitate electron transfer and surface NO removal [21]. C–O will prevent the adsorption of pollutants and reduce the adsorption capacity of the material [22]. Therefore, the decrease of C–O functional group content and the increase of –COO functional group content in the absorbent after hydration treatment are beneficial to denitrification.

**Figure 8:**
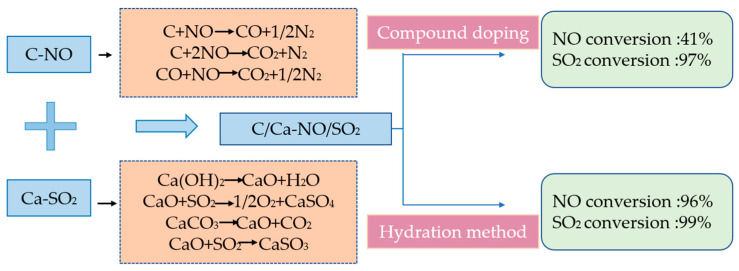
Mechanism of flue gas desulfurization and denitrification in cement kiln based on C–CaO–SiO_2_ coupling enhancement effect.

The desulfurization and denitrification absorbent studied in the current experiment are generally used at a low temperature of about 100 °C, which is not suitable for the high temperature and high dust environment during the formation of cement clinker. At the same time, there is a phenomenon of SO_2_ and NOx competitive adsorption in cement kilns, the adsorption and removal temperature ranges of SO_2_ and NOx are not equal. In this paper, the rice husk ash-carbide slag absorbent is modified. In addition to the effective sulfur fixation of calcium-based raw materials, carbon-based materials are precipitated in the form of CH, CO, H_2_ at a high temperature of 700–900 °C, which react with the oxygen in the surrounding environment, consuming most or even all of the oxygen, quickly establishing a reducing atmosphere locally, and effectively transforming NO. The C–NO reduction reaction is combined with the Ca–SO_2_ sulfur fixation reaction to form a modified absorbent with enhanced C–CaO–SiO_2_ coupling. At 700 °C, the NO conversion reaches 96%, the SO_2_ conversion reaches 99%, and the denitrification efficiency is greatly improved. The hydrated calcium silicate and hydrated calcium aluminosilicate products are formed during the hydration process. The absorbent has a larger specific surface area and more pore structure, and the content of oxygen-containing functional groups is increased, which is beneficial to the desulfurization and denitrification reactions. The desulfurization and denitrification process of cement kiln flue gas based on the coupling enhancement effect of C–CaO–SiO_2_ is shown in Figure 8.

## 4. Conclusions

In this paper, the hydration method is used to control the component, particle surface modification and surface activation of the rice husk ash-carbide slag desulfurization and denitrification integrated absorbent, so that it can have better microscopic characteristics and higher reactivity. Therefore, a highly active composite absorbent with enhanced C–CaO–SiO2 coupling under the working conditions of the cement kiln is prepared, which promotes the reduction and denitrification reaction of nitrogen oxides without reducing the desulfurization efficiency. We conclude as follow:

1. By changing the hydration temperature, hydration time, mass ratio and solid–liquid ratio of rice husk ash/carbide slag, the optimal preparation conditions for desulfurization and denitrification were determined as follows: the hydration temperature is 130 °C, the hydration time is 8 h, the mass ratio of rice husk ash/ carbide slag is 1:1, and the solid-liquid ratio is 1:20.
2. After the absorbent was regulated and surface activated, the reaction temperature window was widened to 700–900 °C. At 700 °C the NO conversion of the modified absorbent is 96% and the SO_2_ conversion is 99%. Compared with that before modification, NO conversion and SO_2_ conversion increased by 44% and 2% respectively. An absorbent based on the combination of rice husk ash and carbide slag was proposed for the first time in this paper, which can achieve simultaneous desulfurization and denitrification in the lower temperature stage (about 700 °C) of cement clinker firing.
3. During the modification process, hydration products such as fibrous calcium silicate and calcium aluminosilicate are formed. The specific surface area of the absorbent becomes larger, which provides more reactive sites, and the content of oxygen-containing functional groups increases. The decrease of ether CO functional group and the increase of carboxy–COO functional group content are beneficial to the denitrification reaction, solving the problem of the unequal temperature range of SO_2_ and NOx adsorption and removal in the cement kiln.

## Figures and Tables

**Table 1:** Composition analysis of rice husk ash (RHA) and carbide slag (CS) (%).

Sample	CaO	SiO_2_	Al_2_O_3_	SO_3_	Fe_2_O_3_	MgO	P_2_O_5_	K_2_O	MnO	Na_2_O	Cl	Loss
RHA	1.60	87.60	0.50	1.03	0.35	1.34	2.86	4.36	0.36	-	-	-
CS	69.81	2.16	1.19	0.79	0.17	0.07	0.02	-	-	0.44	0.27	25.08

**Table 2:** Orthogonal experimental design table for preparation conditions of absorbent.

Sample Number	Hydration Temperature (°C)	Hydration Time (h)	Rice Husk Ash/Carbide Slag Mass Ratio	Solid-Liquid Mass Ratio
H1	70	5	1:1	1:5
H2	70	8	3:1	1:10
H3	70	11	6:1	1:20
H4	100	5	3:1	1:20
H5	100	8	6:1	1:5
H6	100	11	1:1	1:10
H7	130	5	6:1	1:10
H8	130	8	1:1	1:20
H9	130	11	3:1	1:5

**Table 3:** Specific surface area and pore structure characteristics of rice husk ash, carbide slag and modified absorbent.

Sample	BET Surface (m^2^/g)	Pore Volume (cm^3^/g)	Pore Size (nm)
Rice husk ash	135.8	0.03	6.38
Carbide slag	18.6	0.13	22.62
Modified absorbent	142.1	0.22	12.03

**Table 4:** Relative contents of elements on the surface of absorbents.

Sample	C	O	N
RHA	82.62	16.80	0.58
H8	70.09	27.86	2.05

**Table 5:** XPS mapping analysis of relative contents of different functional groups.

Functional Group (%)	Binding Energy (ev)	Relative Content (%)	
		RHA	H8
C–C	284.2 ± 0.2	85.18	84.10
C–O	285.8 ± 0.2	9.69	5.33
–COO	288.5 ± 0.4	5.14	6.98

## Data Availability

The data presented in this study are available on request from the corresponding author.
